# Cooccurrence of Five Pathogenic *Legionella* spp. and Two Free-Living Amoebae Species in a Complete Drinking Water System and Cooling Towers

**DOI:** 10.3390/pathogens10111407

**Published:** 2021-10-30

**Authors:** Alshae Logan-Jackson, Joan B. Rose

**Affiliations:** 1 Department of Microbiology and Molecular Genetics, Michigan State University, East Lansing, MI 48824, USA; 2 Department of Fisheries and Wildlife, Michigan State University, East Lansing, MI 48824, USA; rosejo@msu.edu

**Keywords:** *Legionella*, *L. pneumophila*, *L. micdadei*, *L. anisa*, *L. bozemanii*, *L. longbeachae*, *N. fowleri*, *Acanthamoeba* spp., water supply system, cooling towers

## Abstract

Pathogenic *Legionella* species grow optimally inside free-living amoebae to concentrations that increase risks to those who are exposed. The aim of this study was to screen a complete drinking water system and cooling towers for the occurrence of *Acanthamoeba* spp. and *Naegleria fowleri* and their cooccurrence with *Legionella pneumophila*, *Legionella anisa*, *Legionella micdadei*, *Legionella bozemanii*, and *Legionella longbeachae*. A total of 42 large-volume water samples, including 12 from the reservoir (water source), 24 from two buildings (influents to the buildings and exposure sites (taps)), and six cooling towers were collected and analyzed using droplet digital PCR (ddPCR). *N. fowleri* cooccurred with *L. micdadei* in 76 (32/42) of the water samples. In the building water system, the concentrations of *N. fowleri* and *L. micdadei* ranged from 1.5 to 1.6 Log_10_ gene copies (GC)/100 mL, but the concentrations of species increased in the cooling towers. The data obtained in this study illustrate the ecology of pathogenic *Legionella* species in taps and cooling towers. Investigating *Legionella*’s ecology in drinking and industrial waters will hopefully lead to better control of these pathogenic species in drinking water supply systems and cooling towers.

## 1. Introduction

Free-living amoebae are ubiquitous protozoa that have been found in various natural and engineered water systems, such as surface water, groundwater, drinking water supply systems, hot springs, and cooling towers [1,2,3,4,5,6,7]. Similar to free-living amoebae, *Legionella* spp. are also found in natural water bodies (surface water, groundwater, and hot springs) [8,9,10,11] and human-made systems (swimming pools, drinking water supply systems, and cooling towers) [12,13,14,15,16,17,18,19,20,21]. In their natural and engineered environments, free-living amoebae serve as a host, reservoir, and vehicle of pathogenic bacteria, including *Legionella* species [8,9,10,11,12,13,14,15,16,17,18,19,20,21].

*Acanthamoeba* and *Naegleria* are the two most common genera that are frequently isolated from aquatic environments [12,13,14,15,16] and serve as suitable hosts for *Legionella* species (17). *Legionella* species are associated with free-living amoebae in two developmental stages: the active stage, trophozoite (growth: *Legionella* spp. amplify to high concentrations [22]) and the resisting stage, cysts (survival: intracellular growth enables protection for *Legionella* spp. against chemical disinfectants in the water treatment system [23]). *L. pneumophila,* responsible for Legionnaires’ disease (LD) and Pontiac fever [24,25], has evolved a mechanism to invade the host cell by inducing phagocytosis and can avoid being digested by inhibiting the fusion of phagosomes with lysosomes [26,27]. Additionally, *L. pneumophila* growth within free-living amoebae has been shown to enhance the virulence factors of this species [28,29]. After intracellular amoebae replication of *Legionella* spp., the host cell is lysed, and once in the water column, *Legionella* has been shown to be less susceptible to disinfectants and can amplify to concentrations that are a risk to public health [30,31]. To that end, cooling towers and hot-water taps are key sources of human infections with *Legionella* species [32,33], ultimately posing a risk to human health via inhalation as these exposure sites are aerosol spreading units [34,35,36,37].

Specific species of *Legionella*, *Naegleria*, and *Acanthamoeba* have been known to cause disease in humans [38,39]. For example, *L.* *pneumophila*, *L. anisa*, *L. micdadei*, *L. bozemanii*, and *L. longbeachae* covers most of the incidence of LD [40]. Worldwide, *L. pneumophila*, *L. longbeachae*, and *L. bozemanii* account for 91.5%, 3.9%, and 2.4%, respectively, of LD [40]. Together, *L. micadedi* and *L. anisa* cover 1.8% of LD [40]. *N. fowleri* is the only species of *Naegleria* that causes primary amoebic meningoencephalitis (infection of the brain), a fatal disease of the central nervous system (CNS) [41]. *Acanthamoeba* spp. (9/20) causes cutaneous, ocular, and CNS infections, known as chronic granulomatous amebic encephalitis [39,42]. Because of such species relevance to human disease, the cooccurrence of amoebae and *Legionella* species are warranted further investigation in a complete water supply system.

There are limited studies examining their cooccurrence in a complete drinking water system and cooling towers, collectively. The cooccurrence of *Legionella* spp. and free-living amoebae has been examined solely in a drinking water supply system [43], a hospital water network [44], and cooling towers [45]. Each study focused mainly on the cooccurrence of *L. pneumophila* and various amoebae species by collecting a small volume (1 L or less) of the sample [43,44,45]. It is hypothesized that sampling a small volume of water may not represent the true distribution of pathogenic amoebae and coexisting *Legionella* spp. in the environment. Thus, the present paper is aimed at filling in the knowledge gaps in the distribution of pathogenic amoebae species and *Legionella* spp. by collecting a large volume of bulk water from a drinking water source, building water system, and cooling towers, all served by a groundwater source. Using ddPCR, this study addressed the following objectives (i) identify the cooccurrence of pathogenic *Legionella* spp. (*L. pneumophila*, *L. anisa*, *L. longbeachae*, *L. bozemanii*, and *L. micdadei*) and amoebae species (*N. fowleri* and *Acanthamoeba* spp.) in a complete water supply chain and cooling towers, (ii) examine the difference in occurrence and concentration of *N. fowleri* and *Acanthamoeba* spp. in a groundwater source, which supplied the building water system, and cooling towers, all located in the state of Michigan.

## 2. Results

*N.fowleri* occurrence rate was higher 40% (17/42) than detectable *Acanthamoeba* spp. 19% (8/42) (Table 1). In the drinking water system (RES_IN (influent pipe of the reservoir), RES_EF (effluent pipe of the reservoir), Fa (building one), and ERC (building two)), *Acanthamoeba* spp. only occurred in 8% (3/36) of the samples, while *N. fowleri* occurred in 33% (12/36). *Acanthamoeba* spp. only occurred in building Fa and the cooling towers, while. *N. fowleri* occurred in every sampling site except building Fa (Table 1). Water samples collected from the cooling towers were higher in percentage positives for *Acanthamoeba* spp. 83% (5/6) and *N. fowleri* 83% (5/6) than the drinking water system (Fa, ERC, and RES_IN, and RES_EF) (Table 1). The concentrations of *Acanthamoeba* spp. in building Fa ranged from 1.1 to 1.4 Log_10_ GC/100 mL. The concentrations of *N. fowleri* in the reservoir and building ERC ranged from 1.3 to 1.7 and 1.1 to 1.8 Log_10_ GC/100 mL, respectively (Table 1). The concentrations of *Acanthamoeba* spp. and *N. fowleri* in the cooling towers ranged from 2.0 to 2.5 and 1.3 to 2.4 Log_10_ GC/100 mL, respectively (Table 1).

*L. pneumophila* was detected at every sample location (reservoir, buildings, and the cooling towers), and the concentration ranged from 1.4 to 2.8 log_10_ GC/100 mL (Table 1). The other four *Legionella* species were detected throughout the water supply system, and the concentrations ranged from 1.3 to 2.9 log_10_ GC/100 mL (Table 1). Four *Legionella* species (*L. pneumophila*, *L. micdadei*, *L. bozemanii*, and *L. anisa*) were higher in the cooling towers than in the drinking water system (Table 1). In contrast, *L. longbeachae* concentration remained relatively the same through the drinking water system and cooling towers (Table 1).

**Figure 1:**
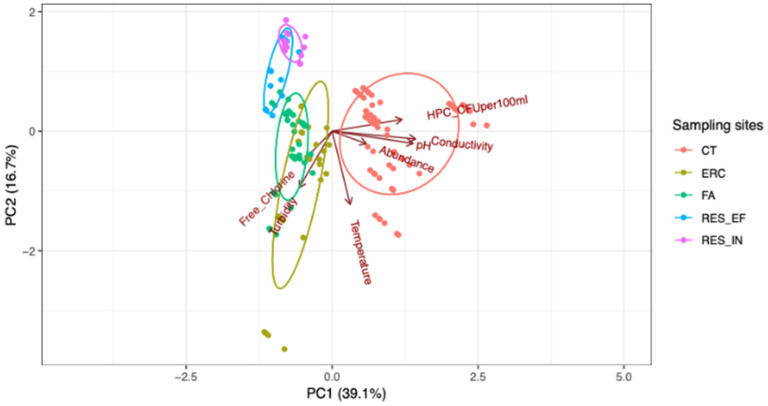
Principal component analysis (PCA) biplot showed a clear separation between water samples collected from the cooling towers relative to those collected from the drinking water system (Fa, ERC, RES_IN, and RES_EF). Each data point represents each species from a particular sampling site (the observation). Samples from five sampling locations, color-coded based on sampling location. The absolute abundance of *Legionella* and amoebae species at each sampling site is depicted in the graph.

Figure 1 shows a clear separation between water samples collected from the cooling towers (CT) and those collected from the drinking water system (RES_IN, RES_EF, Fa, and ERC). The cooling towers’ chemical and microbial parameters are different from the drinking water system (Fa, ERC, and RES_IN, and RES_EF) (Table 2). For instance, the cooling towers are associated with higher bacterial abundance, pH, conductivity, HPC, and lower turbidity, and free chlorine (Table 2). Building Fa and ERC are characterized by free chlorine and turbidity, while reservoir clustering (RES_IN and RES_EF) are associated with lower temperature, abundance, pH, HPC, and conductivity (Table 2).

The chi-squared test of independence showed a significant association between two amoebae species and four pathogenic *Legionella* spp. in all 42 water samples: *Acanthamoeba* spp. and *L. anisa* (χ^2^ = 4.791, *p* = 0.0286), *N. fowleri* and *L. micdadei* (χ^2^ = 4.748, *p* = 0.0293), *N. fowleri* and *L. pneumophila* (χ^2^ = 4.356, *p* = 0.0369), *N. fowleri* and *L. bozemanii* (χ^2^ = 6.645, *p* = 0.0099). *L.* *anisa*-positive samples were observed in the presence or absence of *Acanthamoeba* spp. (Table 3). *L.* *micdadei, L. bozemanii,* and *L.* *pneumophila*-positive samples were also observed in the presence or absence of *N. fowleri* (Table 4, Table 5 and Table 6).

The concordance percentage between the presence of *Acanthamoeba* spp. and *L. anisa* was 9.52%. The absence percentage of both species (*Acanthamoeba* spp. and *L. anisa*) was higher (69.05%) than the presence percentage (9.52%) (Table 3). A total of 78% of the time, either *Acanthamoeba* spp. and *L. anisa* co-exist or were co-absent together (Table 3). *N.* *fowleri* and three specific *Legionella* spp. (*L. micdadei*, *L.* *bozemanii*, and *L.* *pneumophila*) concordance positive percentage was 16.67%, 30.95%, and 30.95%, respectively (Table 4, Table 5 and Table 6). The absence percentage of *N. fowleri* and *L. micdadei*, *L.* *bozemanii*, and *L.* *pneumophila* were higher (52.38%, 38.10%, and 33.33%, respectively) than the positive percentage (Table 4, Table 5 and Table 6). A total of 76% of the time, either *N. fowleri* and *L. micdadei*, co-exist or were co-absent together (Table 4). In the absence of free-living amoebae, *L. anisa*, *L. micdadei*, *L. bozemanii,* and *L. pneumophila* occurred only 11%, 7%, 21%, and 26% of the time (it is hypothesized that amoebae hosts and/or the sloughing of the biofilm may have released *Legionella* species into the water supply system). Thus, the relationship that is observed between these two amoebae and *Legionella* species is driven by the absence percentage of both organisms (Table 3, Table 4, Table 5 and Table 6).

**Figure 2:**
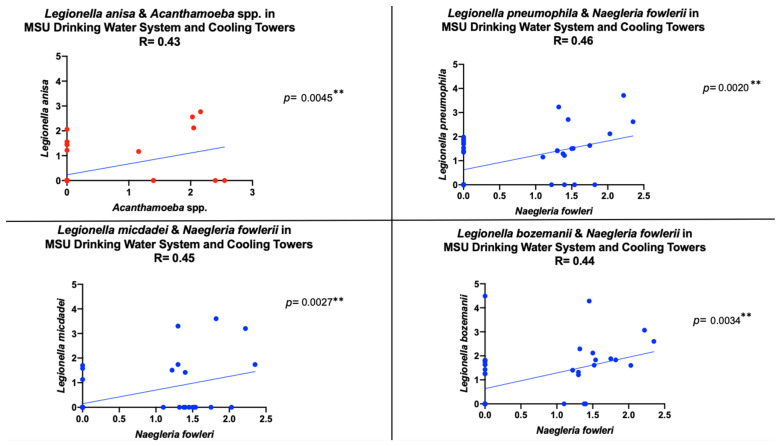
Statistically significant positive Pearson correlation between two amoeba species and four pathogenic *Legionella* spp. in 42 water samples. ** *p* < 0.01.

Figure 2 revealed a statistically significant positive correlation between *Acanthamoeba* spp. and *L. anisa* as well as between *N. fowleri* and *L. micdadei*, *L. pneumophila*, and *L. bozemanii* in all 42 water samples. Out of three influent water samples in the building Fa, *Acanthamoeba* spp. and *L. anisa* cooccurred in one sample (data not shown). Out of six cooling tower samples, *Acanthamoeba* spp. and *L. anisa* cooccurred in three water samples (data not shown). Thus, it is suggested that the relationship between *Acanthamoeba* spp. and *L. anisa* seen in Figure 2 was driven by the absence of both species in the drinking water system (except for building Fa) and the presence of both species in the cooling towers. Out of nine (influent and tap) water samples in building ERC, *N. fowleri* and *L. micdadei* cooccurred in three samples (data not shown). In the cooling tower samples, *N. fowleri* and *L. micdadei* cooccurred in 33% (1/6) of the water samples (data not shown). It is suggested that the relationship between *N. fowleri* and *L. micdadei* seen in Figure 2 was driven by the absence of these species.

## 3. Discussion

This study revealed new quantitative information about the cooccurrence of five pathogenic *Legionella* species and two free-living amoebae species in a complete drinking water system. The chi-square test of independence showed a relationship between four *Legionella* species and two amoebae species. Interestingly, the relationship between *Acanthamoeba* spp. and *L. anisa* was driven by the absence of both species in the influent and effluent of the reservoir, as well as building ERC, and the presence of both species in the cooling towers (data not shown). The relationship between *L. micdadei* absence to *N. fowleri* absence, *L. bozemanii* absence to *N. fowleri* absence, and *L. pneumophila* absence to *N. fowleri* absence is driven by the building water quality and the cooling towers (data not shown). In this study, *Acanthamoeba* spp. and *L. anisa*, *L. micdadei* and *N. fowleri*, *L. bozemanii* and *N. fowleri*, and *L. pneumophila* and *N. fowleri* co-existence and absence occurred in 78%, 76%, 47%, and 42% of the water samples, respectively. The reason for this cooccurrence and collective absence is not completely clear; however, the water chemistry of the water supply system could be the contributing factor that is driving the microbial ecology. For example, the building water system (Fa and ERC) is characterized by free chlorine and turbidity, while the reservoir is associated with lower temperature, pH, HPC, and conductivity. The cooling towers are different from the drinking water system (reservoir and buildings) as they are associated with higher pH, conductivity, and lower free chlorine residual. Previous reports have also shown that *Acanthamoeba* spp. and *N. fowleri* were detected in a drinking water supply system [43] and cooling towers by qPCR [45].

The concentrations of the specific *Legionella* and amoebae species ranged from 1.3 to 2.9 and 1.1 to 2.5 log_10_ GC/100 mL, respectively, in the complete water supply system (from source to taps and cooling towers). The average concentration for both organisms (*Legionella* and amoebae species) was 1.6 log_10_ GC/100 mL. In this study, the cooling towers support high concentrations of *Legionella* and amoebae species relative to the building water system, and this fact is due to the water chemistry in the cooling towers (described above). Cooling towers have been the source of LD outbreaks [46]. This study further demonstrated that the cooling towers are a risk for LD outbreaks as this exposure site had increased concentrations of both *Legionella* species and its host, free-living amoebae. Currently, the commercial monitoring of cooling towers is solely focused on detecting *L. pneumophila* by culture-dependent methods and does not consider its host and vehicle of function, free-living amoebae [46,47,48,49]. Additionally, there are some limitations to the standard culture method [48,50]. For example, the culture method takes up to 10 days for results, it is incapable of enumerating viable but non-culturable cells (VBNC), and it is unable to identify *Legionella* species in a precise manner [50]. However, quantitative assessment using PCR can provide critical results on bacterial and amoebae concentrations to determine if there is amplification at a particular exposure site [51,52]. Digital droplet PCR was very useful and can be used to assess duplex assays for various species, as described in this study. Digital droplet PCR has been reported to show higher sensitivity and specificity for known concentrations of *L. pneumophila* in water samples than real-time quantitative PCR [53]. Additionally, ddPCR has been shown to quantify absolute, low DNA concentration and DNA in a host-pathogen interaction context [54,55]. Given such information, ddPCR was used to assess free-living amoebae as well as intra-amoebae *Legionellae* replication. While there are some limitations to assessing *Legionella* and amoebae host using ddPCR (it is incapable of distinguishing live and dead cells), there is an assumption that if all cells are culturable, the ratio of culture to the molecular method (PCR) is 7 CFU to 3 GC [56]. Thus, there is a need for a rapid, precise test to monitor the cooling towers. There is also a need for a greater comparison examining VBNC cells as well as live and dead cells to address the health risk by using and comparing molecular and culture methods to routinely quantify *Legionella* and amoebae species collected from various exposure sites (taps and cooling towers). A PCR method can serve as an early detection tool to warn the building owner of a potential outbreak, while the culture method can be used for the confirmation of the occurrence and concentration of these important species [51]. Overall, local and federal testing laboratories should incorporate a parallel method (culture and molecular method) to screen for both *Legionella* and host amoebae into their routine screening for taps and especially cooling towers.

The widespread of *N. fowleri* and *Legionella* species in the taps and cooling towers indicates an important health concern. As stated above, the cooling towers have different characteristics from the drinking water system; therefore, it is important to investigate free-living amoebae presence and concentration in a water supply system as they serve as vehicles for bacterial pathogens. In this study, *N. fowleri* was more often detected in the whole water supply system than *Acanthamoeba* spp. It is suggested that the microbial ecology and water chemistry affected the amplification of *N. fowleri* in the complete water supply system (water source, water age, and cooling towers). Consistent with this study, *N. fowleri* was frequently detected relative to *Acanthamoeba* spp. by PCR molecular technique in untreated and treated water [57], tap water [58], and from municipal drinking waters and recreational water sources [59]. This work presented herein demonstrated that *L. micdadei* and *L. bozemanii* were more often related to *N. fowleri* than *Acanthamoeba* spp., and the correlation between these species in the building water system and cooling towers observed separately (data not shown) could be due to the microbes being released into the water column from sloughing of the biofilms. Thus, it is important to identify the ecological factors that favor the presence of *Legionella* to improve its control in the built environment (taps, cooling towers). Several studies have focused on investigating and controlling *Legionella* [60,61,62] without considering the association with these host reservoirs, *Naegleria* and *Acanthamoeba* spp. It is hypothesized that if there is control of free-living amoeba, then one can subsequently control *Legionella* species in a water supply system.

A couple of studies have suggested that the concentrations of *N. fowleri* and *L. pneumophila* amplification in a distribution system may be seasonally influenced via water temperature [63,64]. In this study, the results showed that the detection of free-living amoeba and *Legionella* spp. was not due to ambient temperature. For example, pathogenic *L. micdadei*, *L. bozemanii*, and *N. fowleri* were detected more in a drinking water supply system that was supplied by a groundwater source (supplied by production wells), which is not affected by ambient temperatures such as surface waters. The occurrence of free-living amoeba and *Legionella* spp. may be affected by the built environment (i.e., water age). Water age has been observed to affect the detection of *Legionella* and amoebae species in a distribution system [59,64]. In this study, it is suggested that the increase in water age in the distribution system is associated with an increase in *Legionella* and amoebae species. Thus, water age may play a role in the occurrence of *Legionella* and amoebae species in a building with an increased water age, as seen in the ERC building (water age of 20.8 h) and the cooling towers (water age of 23.2 h).

Since free-living amoebae promote the growth of *Legionella* post-treatment in water systems [22], it is critical to gain an understanding of microbial-amoebae ecological relationships in large, complicated plumbing. The regression analysis (data not shown) revealed in this study showed that *N. fowleri* affects the occurrence of *L.* *micdadei* and *L. bozemanii* in building ERC, furthest away from the reservoir. The regression analysis emphasizes that it is a building issue, and the water quality in building ERC is driving the occurrence of *N. fowlerii*, *L. micdadei*, and *L. bozemanii.* However, the regression suggests that this observation will need to be further elucidated with a more extensive data set. An extensive data set such as increasing the total number of samples, sampling and analyzing the biofilm, and verifying the ddPCR results with DNA sequencing would overcome this study limitation. Overall, this study found a greater association between *N. fowleri* and two *Legionella* species.

## 4. Materials and Methods

### 4.1. Site Location and Sampling

Water samples were collected during July, August, and September of 2019 from the reservoir (influent: RES_IN and effluent: RES_EF), two research buildings (Fa and ERC), and six cooling towers on a large research institution that runs its own water system, all located in the state of Michigan. The reservoir contained two large storage tanks for the untreated (RES_IN) and treated water (RES_EF). The building characteristics (water usage, distance from the reservoir, and building age) are described below. The average monthly water usage (five-year average) and distance from the reservoir for Fa was 172,993 L/month and 4.7 km and for ERC was 738,533 L/month and 19.4 km. At the time of sample collection, the building age for Fa (construction year: 1948) and ERC (construction year 1986) were 71 and 33 years, respectively.

A total of 42 large-volume samples were collected from the RES_IN and RES_EF, two research buildings (Fa and ERC), and six cooling towers. The water supply system was supplied by a groundwater source. At the time of sampling, the groundwater source contained ~20 production wells; these wells drew the groundwater from an aquifer to the influent storage tank. The reservoir had two large water storage tanks: influent (untreated groundwater) and effluent (treated groundwater). Ten liters were collected from each site location (RES_IN and RES_EF), buildings (Fa and ERC), and cooling towers in a carboy containing 10% sodium thiosulfate to neutralize the residual chlorine. Ten liters of grab sample was collected from influent and effluent of the reservoir. For the building influent samples, 10 L grab sampling were collected from both building’s (Fa and ERC) influent sampling port. Building Fa influent pipe contained a sampling port, which was accessible for sample collection. Building ERC did not have a sampling port on the influent pipe; thus, it was decided to sample the nearest valve to the influent pipe, which was an eye-wash station located in a mechanical room, where the influent pipe entered the building. For the potable tap samples, 10 L composite sampling from the cold- and hot-water taps were collected as separate samples to evaluate and compare the water quality of the cold-water taps and the hot-water taps on the first and top floor, respectively. The volume of composite sampling was based on the number of sinks per floor. The points of use locations were sink faucets and showerheads located in bathrooms, locker rooms, and breakrooms. Building Fa had two floors, with two and three sinks on the first and second floors, respectively. Building ERC only had one floor, with 11 sinks and 2 showers. Because building Fa had two sinks on the first floor, 5 L of water was collected from each tap (cold and hot) and composited to make a total of two 10 L large samples for the first floor. This sampling regime was the same for the second floor of Fa and building ERC. Per sampling date, building Fa had a total of five samples, one from the influent sampling location and two composite samples per tap per floor (one cold-water composite sampling and one hot-water composite sampling/floor). Building ERC had a total of three samples: one for the influent sampling location and two composite samples (cold- and hot-water). There were several cooling towers on the research institution, but only six from the power plant on campus were sampled. There were sampling replicates per sampling site, and it is as follows: the reservoir and cooling towers were sampled six different times on different days, and each building was sampled three different times on different days. Thus, there were a total of 12 samples collected from the reservoir: influent (6 from RES_IN) and effluent (six from RES_EF), 6 from the cooling towers, and 24 from the buildings (15 from building Fa, nine from building ERC). Each water sample was processed immediately after collection.

### 4.2. Chemical and Physical Analyses

A 300 mL sample was collected separately from the large volume (mentioned above) for chemical and physical parameters. The water samples were analyzed for temperature and chlorine residuals (total and free) onsite using calibrated thermometers and the Test Kit Pocket Colorimeter II (HACH, Loveland, CO, USA) according to the manufacturer’s instructions. After sampling, conductivity, pH, and turbidity were measured offsite according to the manufacturers’ instructions using a Russell RL060C Portable Conductivity Meter (Thermo Scientific, Waltham, MA, USA), UltraBasic pH meter (Denver Instrument, Bohemia, NY, USA), and a Turbidity Meter code 1970-EPA (LaMotte Company, Chestertown, MD, USA).

### 4.3. Microbiological Analysis

All samples were transported on ice to the laboratory and processed the same day. All samples collected for this study were tested for heterotrophic plate count (HPC) analyses using membrane filters (47 mm diameter, 0.45 μm pore size) (PALL Corporation, Port Washington, NY, USA) on m-HPC agar (Becton, Dickinson and Company, Difco™, Detroit, MI, USA). All plates were incubated for 48 ± 2 h at 35–37 °C, then enumerated for colony-forming units (CFU).

### 4.4. Water Sample Processing and DNA Extraction

The 10 L water samples were processed using a single-use Asahi REXEED-25S dialysis filter (Dial Medical Supply, Chester Springs, PA, USA). The Asahi REXEED filter was pretreated with 0.01% of sodium hexametaphosphate (used to trap microbial material onto each ultrafilter) and was used in a dead-end mode. After filtration, a high-pressure single-use elution fluid canister (Innovaprep LLC, Drexel, MO, USA) was used to concentrate the 10 L large volume to ∼50 mL. The 50 mL sample was aliquoted into 5–10 mL samples.

### 4.5. Molecular Analysis

For each sample, one 10 mL (remaining sample was stored at −80 °C) subsample was further filtered through a 47 mm, 0.45 μm polycarbonate filter (Whatman, Kent, U.K.) for DNA extraction. The polycarbonate filter was folded into a 1/8 shape with the contents of the filter folded to the inside. The filter was then transferred to a 2.0 mL polypropylene screw cap tube (VWR, Radnor, PA, USA), which contained 0.3 g of 212–300 μm acid-washed glass beads (Sigma, St. Louis, MO, USA) for a crude DNA extraction procedure, which is described below. DNA extraction was performed by adding 590 μL of AE buffer (Qiagen, Redwood City, CA, USA) to the samples to assist with lysing the cell membrane, and then bead milling using a FastPrep-24™ 5G Instrument MP Biomedicals (VWR, Radnor, PA, USA). Samples were bead-milled at 6000 rpm for one minute, then followed by centrifugation at 12,000× *g* for an additional minute (60 s). Approximately 400 μL of the supernatant was transferred to a new clean microcentrifuge tube and centrifuged at 12,000× *g* for an additional three minutes to pellet any remaining debris. Approximately 350 uL of extracted nucleic acid was eluted into a final clean microcentrifuge tube. Sixty microliters of the eluted volume were then aliquoted into several microcentrifuge tubes (∼five extraction replicates per sample) for storage at −80 °C to reduce the need for several freeze/thaw cycles. One aliquot per water sample was later used for PCR analysis (samples were held at −80 °C for up to 30 days before analysis).

### 4.6. Molecular Analysis of Acanthamoeba spp., N. fowleri, General Legionella spp., and Four Pathogenic Legionella Species

Droplet digital PCR (Bio-Rad Laboratories, Hercules, CA, USA) technology was performed according to the manufacturer’s instructions to analyze each sample for two amoebae species (*Acanthamoeba* spp. *N. fowleri*) and five pathogenic *Legionella* spp. (*L. pneumophila*, *L. anisa*, *L. micdadei*, *L. bozemanii*, and *L. longbeachae*). The primers and probes listed in this study are listed in Table 7. Duplex reactions were performed for four separate assays: the first assay consisted of *Legionella* spp. (data not shown) and *L. pneumophila*, the second assay comprised of *L. micdadei* and *L. anisa*, the third assay consisted of *L. bozemanii* and *L. longbeachae*, and the fourth assay contained *Acanthamoeba* spp. *N. fowler* (Table 7). All primers and probes were ordered from Eurofins Genomics Co., (Louisville, KY, USA).

Positive controls using genomic DNA from amoebae, *A.* castellani strain Neff (ATCC No. 30010) and *N. fowleri* (ATCC No. 30174) and bacteria, *L. pneumophila* (ATCC No. No. 33152)*, L. anisa* (ATCC No. 35292)*, L. micdadei* (ATCC No. 33218)*, L. bozemanii* (ATCC No. 33217)*,* and *L. longbeachae* (ATCC No. 33462), were obtained from American Type Culture Collection (ATCC, Manassas, VA, USA). Each control (positive and negative) was run with each ddPCR plate. As part of the quality control, sample results were only considered for analysis when the reader accepted 10,000 or more droplets. Per assay, there was one ddPCR run with three biological (sample collection replicates) and three technical replicates (same extraction) tested. Biological and technical replicates were run for each sample to determine if there was a wide variation among sampling days and whether the assay results were reproducible, respectively.

Droplet digital PCR technology was performed according to the manufacturer’s instructions. In brief, the reaction mixture consisted of 2X supermix (no dUTP) (Bio-Rad Laboratories, Hercules, CA, USA), 900nM forward and reverse primers, and 250 nM for each organism (bacteria and amoebae) probe (Eurofins Genomics Co., Lousiville, KY, USA), and up to 330 ng of DNA template, to a final volume of 20 μL. Twenty microliters of the sample reaction mixtures were loaded into a DG8 cartridge (Bio-Rad Laboratories, Hercules, CA, USA), followed by 70 μL of droplet generator oil (Bio-Rad Laboratories, CA, USA). Droplets were generated using the QX200™ Droplet Generator Bio-Rad) and transfer of emulsified samples to a ddPCR 96-well plate (semi-skirted) and was performed according to manufacturer’s instructions (Instruction Manual, QX200™ Droplet Generator—Bio-Rad). The ddPCR plate was sealed with pierceable foil heat seals using a PX1^TM^ PCR Plate Sealer (Bio-Rad, Laboratories, CA, USA). The plate was amplified using a Benchmark TC9639 thermal cycler (Benchmark Scientific Inc, Sayreville, NJ, USA). The cycling protocol was as follows: 95 °C for 10 min, followed by 40 cycles of 94 °C for 30 sec and 57 °C for 1 min with a final 10 min cycle at 98 °C for 10 min. After endpoint amplification, droplets were read using a QX200 droplet reader (Bio-Rad QX200^TM^ Droplet Digital^TM^ PCR System, Hecules, CA, USA). Two negative controls, a filtration blank (phosphate-buffered water) and a non-template control (molecular grade water), were run with each ddPCR plate. Negative and positive controls were used to determine contamination (if any) and the efficiency of the assay.

### 4.7. Statistical Analysis

Statistical analysis was performed in GraphPad Prism 8 software (GraphPad Software, San Diego, CA, USA). Contingency tables, chi-squared tests, and Pearson correlation were used to evaluate the associations between the occurrence of *Legionella* spp. and free-living amoebae. Additionally, the principal component analysis was also conducted to assess the associations between the cooccurrence of both organisms and different water quality parameters.

Sample concentrations were transformed from (GC)/100 mL into Log_10_ GC/100 mL. Biological data were expressed as geometric means, and chemical data were shown as arithmetic means with standard deviation [52]. Statistical results were interpreted at the level of significance *p* < 0.05.

## 5. Conclusions

Most of the public health agencies and guidance documents have water management programs that are focused on the risk of *Legionella*, without considering their host, *Naegleria*, and *Acanthamoeba* spp. Using ddPCR, *N. fowleri* was detected more often in a drinking water supply system and cooling towers. The chi-squared test showed that *N. fowleri* significantly cooccurred with three pathogenic *Legionella* spp. (*L.* *pneumophila*, *L.* *micdadei*, and *L. bozemanii*) in a drinking water supply system and cooling towers. In this study, *L. micdadei* and *L. bozemanii* were more often related to *N. fowleri* than *Acanthamoeba* species. Thus, by examining large volume (10 L), water ultrafiltrate concentrates from the groundwater source to exposure sites (taps and cooling towers) using ddPCR allowed for the detection of individual-specific *Legionella* and *N. fowleri*. Most importantly, the widespread of *N. fowleri* and *Legionella* species in the taps and cooling towers indicates an important health concern.

## Figures and Tables

**Table 1:** Concentrations of amoebae and *Legionella* species increased in water samples collected from the cooling towers (CT) than in the drinking water system (RES_IN, RES_EF, Fa, and ERC). Influent, hot-, and cold-water samples were nested together for buildings Fa and ERC.

Free-Living Amoebae	Site Location				
	Res_In	Res_EF	Fa	ERC	CT
*Acanthamoeba* spp. (%)	0/6 (0%)	0/6 (0%)	3/15 (20%)	0/9 (0%)	5/6 (83%)
*Acanthamoeba* spp. Min, and Max Geomean (Log_10_ GC/100 mL)	ND	ND	1.1, 1.4	ND	2.0, 2.5
*N.fowleri* (%)	5/6 (83%)	1/6 (16%)	0/15 (0%)	6/9 (66%)	5/6 (83%)
*N.fowleri* Min, and Max Geomean (Log_10_ GC/100 mL)	1.3, 1.7	1.5 ^a^	ND	1.1, 1.8	1.3, 2.4
*Legionella* Species					
*Legionella* spp. (23S rRNA) (%)	(6/6) (100%)	(6/6) (100%)	15/15 (100%)	9/9 (100%)	6/6 (100%)
*Legionella* spp. (23S rRNA) Geomean (Log_10_ GC/100 mL)	3.1	2.7	2.3	4.3	4.5
*L. pneumophila* (%)	5/6 (83%)	5/6 (83%)	6/15 (40%)	3/9 (33%)	5/6 (83%)
*L. pneumophila* Geomean (Log_10_ GC/100 mL)	1.6	1.8	1.6	1.4	2.8
*L. micdadei* (%)	1/6 (17%)	0/6 (0%)	3/15 (20%)	4/9 (44%)	2/6 (33%)
*L. micdadei* Geomean (Log_10_ GC/100 mL)	1.5	ND	1.5	1.6	2.4
*L. bozemanii* (%)	4/6 (67%)	(6/6) (100%)	2/15 (13%)	4/9 (44%)	6/6 (100%)
*L. bozemanii* Geomean (Log_10_ GC/100 mL)	1.5	1.7	1.4	1.5	2.9
*L. longbeachae* (%)	0/6 (0%)	0/6 (0%)	8/15 (53%)	4/9 (44%)	3/6 (50%)
*L. longbeachae* Geomean (Log_10_ GC/100 mL)	ND	ND	1.4	1.4	1.5
*L. anisa* (%)	0/6 (0%)	0/6 (0%)	5/15 (33%)	0/6 (0%)	4/6 (67%)
*L. anisa* Geomean (Log_10_ GC/100 mL)	ND	ND	1.4	ND	2.1

^a^ Only value detected. ND: no data.

**Table 2:** Water age impacted the microbial and chemical parameters in the building water system (Fa and ERC) and the cooling towers. Sample collection dates are as follows: reservoir (RES_IN and RES_EF), July 15th, 23rd, 29th and August 6th, 13th, and 20th; influent, hot-, and cold-water in building Fa, August 12th, and September 3rd, and 16th; influent, hot-, and cold-water in building ERC, August 19th, and September 9th, and 23rd; cooling towers, July 25th, 31st, and August 7th, 14th, and 21st. Sample replicates were collected on different days. Water age in the table is presented as hours, and it is defined as the time from the wells to the influent pipe, the influent pipe to the effluent pipe, the effluent pipe to the influent pipe of buildings, and the cooling towers. The dashes (–) indicate the same time as each respective building influent. Water quality parameters for building influent and potable water samples (hot-, and cold-water) are presented individually.

Temperature (°C)	Total Chlorine (mg/L)	Free Chlorine (mg/L)	Turbidity NTU	pH	Conductivity (mS)	HPC (CFU/100 mL)	Water Age (h)
**RES_IN (N = 6)**							
12.1	0	0	4.1	7.2	851	3.52 × 10^1^	4.5
**RES_EF (N = 6)**							
11.9	0.64	0.33	3.85	7.2	855	2.10 × 10^0^	3.4
**Building F Influent (N = 3)**							
26.8	0.41	0.35	8.4	7.3	897	8.57 × 10^4^	9.2
**Building Fa 1st Floor Cold; N = 3** **(Hot Taps; N = 3)**							
26.7 (28.6)	0.16 (0.04)	0.14 (0.02)	3.06 (0.53)	7.2 (7.1)	867 (815)	1.02 × 10^4^ (7.3 × 10^3^)	–
**Building Fa 2nd Floor Cold; N = 3** **(Hot Taps; N = 3)**							
26.8 (28.8)	0.05 (0.02)	0.03 (0)	3.37 (0.67)	7.0 (6.9)	856 (822)	2.00 × 10^4^ (3.15 × 10^3^)	–
**Building ERC Influent (N = 3)**							
31.5	0.31	0.20	12.5	7.4	883	4.32 × 10^5^	20.8
**Building ERC 1st Floor Cold (N = 3)** **(Hot Taps; N = 3)**							
23.5 (24.5)	0.09 (0.04)	0.03 (0)	5.97 (6.27)	7.6 (7.5)	866 (847)	4.38 × 10^5^ (6.80 × 10^5^)	–
**Cooling Towers (N = 6)**							
25.3	0.49	0.08	1.94	8.2	2564	2.35 × 10^7^	23.2

**Table 3:** A total of 78% (33/42) of the water samples *Acanthamoeba* spp. and *L. anisa* co-exist or co-absent together.

^a^*p* Value = 0.0286 *	*L. anisa* Present	*L. anisa* Absent	Total
*Acanthamoeba* spp. Present	4 (9.52%)	4 (9.52%)	8
*Acanthamoeba* spp. Absent	5 (11.90%)	29 (69.05%)	34
Total	9 (21.42%)	33 (78.57%)	42

^a^ Strong *p*-value is ≤ 0.05. The asterisks (*) below represent the statistical significance between *Acanthamoeba* spp. and *L. anisa*; *p* = 0.0286; chi-square test.

**Table 4:** A total of 76% (32/42) of the water samples *N. fowleri* and *L. micdadei* co-exist or co-absent together.

^a^*p* Value = 0.0293 *	*L. micdadei* Present	*L. micdadei* Absent	Total
*N.fowleri* Present	7 (16.67%)	10 (23.81%)	17
*N.fowleri* Absent	3 (7.14%)	22 (52.38%)	25
Total	10 (23.8%)	32 (76.19%)	42

^a^ Strong *p*-value is ≤ 0.05. The asterisks (*) below represent the statistical significance between *N. fowleri* and *L. micdadei*; *p* = 0.0293; chi-square test.

**Table 5:** A total of 47% (20/42) of the water samples *N. fowleri* and *L. bozemanii* co-exist or co-absent together.

^a^*p* Value = 0.0099 **	*L. bozemanii* Present	*L. bozemanii* Absent	Total
*N.fowleri* Present	13 (30.95%)	4 (9.52%)	17
*N.fowleri* Absent	9 (21.43%)	16 (38.10%)	25
Total	22 (52.38%)	20 (47.61%)	42

^a^ Strong *p*-value is ≤ 0.05. The asterisks (**) below represent the statistical significance between *N. fowleri* and *L. bozemanii*; *p* = 0.0099; chi-square test.

**Table 6:** A total of 42% (18/42) of the water samples *N. fowleri* and *L. pneumophila* co-exist or co-absent together.

^a^*p* Value = 0.0369 *	*L. pneumophila* Present	*L. pneumophila* Absent	Total
*N.fowleri* Present	13 (30.95%)	4 (9.52%)	17
*N.fowleri* Absent	11 (26.19%)	14 (33.33%)	25
Total	24 (57.14%)	18 (42.85%)	42

^a^ Strong *p*-value is ≤ 0.05. The asterisks (*) below represent the statistical significance between *N. fowleri* and *L. pneumophila; p* = 0.0369; chi-square test.

**Table 7:** Free-living amoebae primers and probes. 23S pan-*Legionella* is conserved by all species of *Legionella*, but the probes were uniquely designed to specifically identify each of these species: *L. micdadei*, *L. anisa*, *L. bozemanii*, and *L. longbeachae*. All primers and probes were designed and validated previously by the authors listed in the references below.

Target Species	Primer/Probe Name	Primer/Probe Sequence	Amplicon Length (bp)	Reference
*Acanthamoeba* spp.	18S rRNAF 18S rRNAR 18S rRNAP	5′-CGACCAGCGATTAGGAGACG-3′ 5′-CCGACGCCAAGGACGAC-3′ 5′-FAM-TGAATACAAAACACCACCATCGGCGC-BHQ1-3′	63	[65]
*N. fowleri*	ITSF ITSR ITSP	5′-GTGAAAACCTTTTTTCCATTTACA-3′ 5′-AAATAAAAGATTGACCATTTGAAA-3′ 5′-HEX-GTGGCCCACGACAGCTTT-BHQ1-3′	69	[66,67]
*Legionella species*	23SF 23SR 23SP	5′-CCCATGAAGCCCGTTGAA-3′ 5′-ACAATCAGCCAATTAGTACGAG TTAGC-3′ 5′-HEX-TCCACACCTCGCCTATCAACGTCGTAGT-BHQ1-3′	92	[68]
*L. pneumophila*	mipF mipR mipP	5′-AAAGGCATGCAAGACGCTATG-3′ 5′-GAAACTTGTTAAGAACGTCTTTCATTTG-3′ 5′-FAM-TGGCGCTCAATTGGCTTTAACCGA-BHQ1-3′	78	[68]
*L. micdadei* *L. anisa* *L. bozemanii* *L. longbeachae*	Pan-*Legionella* F Pan-*Legionella* R LmicdadeiP LanisaP Lbozemanii LlongbeachaeP	5′-GTACTAATTGGCTGATTGTCTTG-3′ 5′-TTCACTTCTGAGTTCGAGATGG-3′ 5′-FAM-AGCTGATTGGTTAATAGCCCAATCGG-BHQ1-3′ 5′-HEX-CTCAACCTACGCAGAACTACTTGAGG-BHQ1-3′ 5′-FAM-TACGCCCATTCATCATGCAAACCAGnT-BHQ1-3′ 5′-HEX-CTGAGTATCATGCCAATAATGCGCGC-BHQ1-3′	Not available	[69]

## Data Availability

Not applicable.
